# Sex chromosome complement contributes to sex differences in coxsackievirus B3 but not influenza A virus pathogenesis

**DOI:** 10.1186/2042-6410-2-8

**Published:** 2011-08-01

**Authors:** Dionne P Robinson, Sally A Huber, Mohamad Moussawi, Brian Roberts, Cory Teuscher, Rebecca Watkins, Arthur P Arnold, Sabra L Klein

**Affiliations:** 1 The W Harry Feinstone Department of Molecular Microbiology and Immunology, The Johns Hopkins Bloomberg School of Public Health, Baltimore, MD 21205, USA; 2 Department of Pathology, University of Vermont, Burlington, VT 05446, USA; 3 Department of Medicine, University of Vermont, Burlington, VT 05446, USA; 4 Department of Integrative Biology and Physiology, Laboratory of Neuroendocrinology of the Brain Research Institute, University of California, Los Angeles, CA 90095, USA

## Abstract

**Background:**

Both coxsackievirus B3 (CVB3) and influenza A virus (IAV; H1N1) produce sexually dimorphic infections in C57BL/6 mice. Gonadal steroids can modulate sex differences in response to both viruses. Here, the effect of sex chromosomal complement in response to viral infection was evaluated using four core genotypes (FCG) mice, where the *Sry *gene is deleted from the Y chromosome, and in some mice is inserted into an autosomal chromosome. This results in four genotypes: XX or XY gonadal females (XXF and XYF), and XX or XY gonadal males (XXM and XYM). The FCG model permits evaluation of the impact of the sex chromosome complement independent of the gonadal phenotype.

**Methods:**

Wild-type (WT) male and female C57BL/6 mice were assigned to remain intact or be gonadectomized (Gdx) and all FCG mice on a C57BL/6 background were Gdx. Mice were infected with either CVB3 or mouse-adapted IAV, A/Puerto Rico/8/1934 (PR8), and monitored for changes in immunity, virus titers, morbidity, or mortality.

**Results:**

In CVB3 infection, mortality was increased in WT males compared to females and males developed more severe cardiac inflammation. Gonadectomy suppressed male, but increased female, susceptibility to CVB3. Infection with IAV resulted in greater morbidity and mortality in WT females compared with males and this sex difference was significantly reduced by gonadectomy of male and female mice. In Gdx FCG mice infected with CVB3, XY mice were less susceptible than XX mice. Protection correlated with increased CD4+ forkhead box P3 (FoxP3)+ T regulatory (Treg) cell activation in these animals. Neither CD4+ interferon (IFN)γ (T helper 1 (Th1)) nor CD4+ interleukin (IL)-4+ (Th2) responses differed among the FCG mice during CVB3 infection. Infection of Gdx FCG mice revealed no effect of sex chromosome complement on morbidity or mortality following IAV infection.

**Conclusions:**

These studies indicate that sex chromosome complement can influence pathogenicity of some, but not all, viruses.

## Background

Males and females differ in their susceptibility to a variety of viral pathogens [1]. The mechanisms for this sexual dimorphism are complex and can involve hormonal, behavioral and genetic factors. Females typically generate enhanced immune responses compared to males [2-4], which can accelerate virus clearance and reduce virus load, but can be detrimental by causing immunopathology or the development of autoimmune disease. Immunity to viruses varies with changes in hormone concentrations caused by natural fluctuations over the menstrual or estrous cycle, contraception use, and pregnancy [5]. Estradiol influences multiple aspects of both innate and adaptive immunity including: enhancing dendritic cell differentiation and antigen presentation [6], suppressing lymphoid cell expression of tumor necrosis factor (TNF)α and interleukin (IL)-6 [7,8], increasing lymphoid cell production of IL-4 and interferon (IFN)γ [9-11], increasing immunoglobulin synthesis [12], inhibiting B cell apoptosis [13], suppressing T and B cell lymphopoiesis [14], and promoting forkhead box P3 (FoxP3)+ T regulatory cell development [15-17]. In contrast, androgens are usually immunosuppressive and inhibit both humoral and cellular immunity, including natural killer cell activity [18,19].

Although direct effects of gonadal steroids cause many sex differences in physiology, some sex differences are also caused by the inherent imbalance in the expression of genes encoded on the X and Y chromosomes [20-22]. Many genes on the X chromosome regulate immune function and play an important role in modulating sex differences in the development of immune-related diseases [23]. These immune-related genes code for proteins ranging from pattern recognition receptors (for example, *Tlr7 *and *Tlr8*) to cytokine receptors (for example, *Il2rg *and *Il13ra2*) and transcriptional factors (for example, *Foxp3*) [24]. As a result, X-linked immunodeficiencies are more prevalent in males. However, autoimmune diseases occur more frequently in females, probably as a result of differences in effects of gonadal hormones and sex chromosome genes [23,25].

The *Sry *gene on the Y chromosome causes testes formation and testosterone synthesis leading to male-typical development of many phenotypes, whereas the absence of *Sry *results in ovaries and female-typical development [26]. The 'four core genotypes' (FCG) mouse model has been developed to investigate the impact of sex chromosomes (XX vs XY) and gonadal type (testes vs ovaries) on phenotypes. In FCG mice, *Sry *is deleted from the Y chromosome and an *Sry *transgene is inserted onto an autosome. Deletion of the *Sry *gene results in XYminus (XY-) mice that are gonadal females (that is, with ovaries) whereas insertion of the *Sry *transgene onto an autosome in XX or XY- mice (XX*Sry *and XY-*Sry*) results in gonadal males (that is, with testes). The FCG are: XX gonadal females (XXF), XY- gonadal females (XYF), XX*Sry *gonadal males (XXM) and XY-*Sry *gonadal males (XYM). Depletion of gonadal steroids by gonadectomy of FCG mice unmasks profound effects of sex chromosome complement on behavior, brain function, renal function, and susceptibility to autoimmune disease [22,27]. In experimental autoimmune encephalitis (EAE) and lupus, for example, the presence of the XX sex chromosome complement worsens disease progression, relative to that in XY mice, and results in decreased production of IL-4, IL-5, and IL-13, but increased IL-13Rα2 expression on dendritic cells [27].

Whether sex chromosome complement modulates sex differences in response to viruses has not been examined. Sexual dimorphism in picornavirus infections, including coxsackievirus B3 (CVB3), in mice is well documented with males showing more severe disease than females [28-30]. In contrast, influenza A virus (IAV) infection is more severe in females than males [31,32]. Much of the sexual dimorphism in the outcome of infection with either CVB3 or IAV depends upon the effects of gonadal hormones on immune responses to viral infection [1]. Whether sex chromosome complement also contributes to sex differences in response to viruses was explored in this study. These studies demonstrate that sex chromosome complement contributes to the severity of disease caused by CVB3 but not IAV suggesting that sex chromosomes can impact susceptibility to some but not all viral infections.

## Methods

### Animals

Adult wild-type (WT) male and female C57BL/6 mice were purchased either from NCI (Frederick, MD, USA) or Jackson Laboratories (Bar Harbor, ME, USA). Mice were age matched to the FCG mice in our facilities. FCG mice backcrossed to a Jackson Labs C57BL/6 background for more than 15 generations were used to obtain litters consisting of XXF, XYF, XXM, and XYM. All experimental FCG mice were bred at UCLA and then sent to the University of Vermont or Johns Hopkins University for viral infection. Mice were maintained at five per microisolator cage under standard housing conditions with a 14:10 light/dark cycle and *ad libitum *access to food and water. All experiments were approved by either the Johns Hopkins University or University of Vermont Animal Care and Use Committee and conducted using approved biosafety level 2 practices and procedures.

### Gonadectomy

All FCG (n = 6-9 per group) and half of the WT animals (n = 7-10 per group) were gonadectomized (Gdx) to remove concurrent effects of sex steroids, which can mask effects of sex chromosomes [33]. For bilateral gonadectomy, females and males were anesthetized with an intramuscular injection of a ketamine (80 mg/kg)/xylazine (6 mg/kg) cocktail (Phoenix Pharmaceutical, St Joseph, MO, USA) and the testes were removed from males and the ovaries from females using aseptic technique as described previously [34-36]. Animals were sutured and given several weeks to recover from surgery. All mice were gonadectomized at 8-10 weeks of age. Surgery was performed at UCLA for CVB3 studies and at Johns Hopkins for IAV studies.

### CVB3 infection

The H3 variant of CVB3 was made from an infectious cDNA clone as described previously [37]. Mice were infected at 15-25 weeks of age with 100 plaque-forming units (PFU) of virus intraperitoneally in 0.5 ml phosphate-buffered saline (PBS) and killed 7 days later.

### IAV infection

The mouse-adapted IAV, A/Puerto Rico/8/1934 (PR8; H1N1; courtesy of Maryna C Eichelberger at the Food and Drug Administration), was used for inoculation. Male and female mice (n = 6-10 per experimental group) were infected at 18-22 weeks of age. Mice were anesthetized with ketamine-xylazine and inoculated intranasally with 10^2 ^50% tissue culture infective dose (TCID_50_) of PR8 in Dulbecco's modified Eagle medium. Body mass, rectal temperature, and mortality were monitored daily for 21 days.

### Organ CVB3 titers

Hearts were aseptically removed from the animals, weighed, homogenized in RPMI 1640 medium containing 5% fetal bovine serum (FBS), L-glutamine, streptomycin and penicillin. Cellular debris was removed by centrifugation at 300 *g *for 10 min. Supernatants were diluted serially using tenfold dilutions and tittered on Hela cell monolayers by the plaque-forming assay [38].

### Histology

Tissue was fixed in 10% buffered formalin for 48 h, paraffin embedded, sectioned, and stained by hematoxylin and eosin. Slides were coded and read blindly using a 0 to 4 scale as published previously [39].

### Antibody depletion of T cells *in vivo*

Mice were injected intraperitoneally with 100 μg monoclonal anti-CD3 antibody (clone 17-A2) or rat IgG2b (clone A95-1) in 0.5 ml PBS on day -3 and +1 relative to injection of virus. T cell depletion was determined by flow cytometry of spleen cells as described below.

### Flow cytometry

CD4+IFNγ+ cells were determined by intracellular cytokine staining [39]. 10^5 ^spleen cells were cultured for 4 h in medium containing 10 μg of Brefeldin A (BFA; Sigma, St. Louis, MO), 50 ng/ml phorbol myristate acetate (PMA; Sigma), and 500 ng/ml ionomycin (Sigma); washed; labeled with a 1:100 dilution of Alexa 647 anti-CD4 (clone GK1.5) or Alexa 647 rat IgG2b (clone A95-1); fixed in 2% paraformaldehyde; permeabilized with 0.5% saponin; and labeled with 1:100 dilutions of PE anti-IFNγ (clone XMG1.2) and Alexa647 anti-IL4 (clone 11B11) or PE and Alexa647 rat IgG1 (clone R3-34). All antibodies were from BD Biosciences/Pharmingen (Franklin Lakes, NJ, USA). T regulatory cells were identified using the Mouse T Regulatory Cell Staining Kit (eBioscience, San Diego, CA, USA) according to manufacturer's directions. Cells were analyzed using a BD LSR II flow cytometer with a single excitation wavelength (488 nm) and a band filter for PE (575 nm). The excitation wavelength for Alexa 647 is 643 nm and a band filter of 660/20 nm. The cell population was classified for cell size (forward scatter) and complexity (side scatter). At least 10,000 cells were evaluated. Positive staining was determined relative to isotype controls.

### Statistical analyses

Survival following infection was compared among experimental groups using log rank sum and χ^2 ^analyses. Other dependent measures were analyzed using two-way analysis of variance (ANOVA) or multivariate ANOVA (MANOVA) with sex/hormone status/sex chromosome complement and days post inoculation as the independent variables. Significant interactions were further analyzed using planned comparisons or the Tukey method for pairwise multiple comparisons. Mean differences were considered statistically significant if *P *< 0.05.

## Results

### Sex chromosome complement impacts susceptibility to CVB3 infection

**Figure 1:**
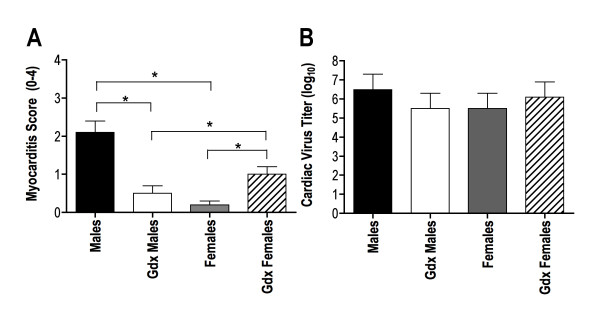
**Myocarditis and cardiac virus titers in intact and gonadectomized (Gdx) male and female C57BL/6 mice infected with coxsackievirus B3 (CVB3)**. Male and female C57BL/6 mice were injected intraperitoneally with 100 PFU CVB3 and surviving mice were killed 7 days later. Half of the animals were gonadectomized 4-6 weeks prior to infection. Hearts were removed, divided and either homogenized for determining virus by the plaque-forming assay or fixed in formalin, paraffin embedded, sectioned and stained by hematoxylin and eosin. **(a) **Mean ± SEM myocarditis score based on a 0-4 scale with 0 = no inflammation; 1 = 1-10 lesions/section; 2 = 11-20 lesions/section; 3 = 21-40 lesions/section and 4 = ≥ 41 lesions/section [39]; and **(b) **virus titers given as mean ± SEM plaque-forming units (PFU) log_10_/g heart tissue of 4-7 mice/group. *Significantly different at *P *< 0.05.

Gonadally intact, WT male and female C57BL/6 mice were infected with CVB3 and by 7 days post infection, 5/10 WT male and 0/10 WT female mice died or were moribund and required killing (χ^2^, *P *< 0.01). WT male C57BL/6 mice develop significantly greater myocarditis than WT female mice (two-way ANOVA sex effect *P *< 0.005), but had similar cardiac virus titers (Figure 1). Gonadectomy of WT male mice reduced CVB3 pathogenicity, whereas gonadectomy of WT female mice increased CVB3 pathogenicity and in the absence of sex hormones, Gdx males became less susceptible to myocarditis than Gdx females (Figure 1a; two-way ANOVA sex × gonadal status effect *P *< 0.00005). This study demonstrates that sex hormones are primarily involved in myocarditis susceptibility in males, whereas sex hormones in females suppress myocarditis susceptibility. In contrast, no significant differences were observed in cardiac virus titers between intact and Gdx animals or between WT male and WT female mice, indicating that pathology is not directly related to CVB3 infection of the myocardium (Figure 1b).

**Figure 2:**
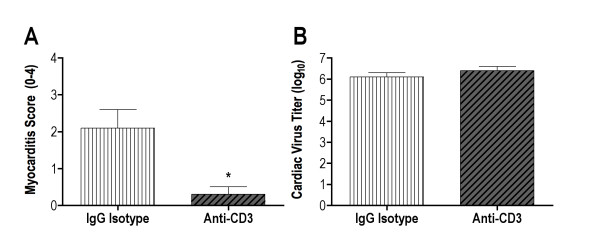
**T lymphocyte depletion protects against coxsackievirus B3 (CVB3) induced myocarditis**. Intact C57BL/6 male mice were injected intraperitoneally with 100 μg monoclonal anti-CD3 antibody or rat IgG2b isotype immunoglobulin on days -3 and +1 relative to infection with CVB3 (100 plaque-forming units (PFU)). Surviving mice were killed 7 days after infection and evaluated for **(a) **myocarditis using a 0-4 histology scale and **(b) **cardiac virus titers (PFU log10/g tissue). Results represent mean ± SEM of 5-7 mice/group. *Significantly different than IgG isotype treated group at *P *< 0.01.

T cells are crucial to CVB3 pathogenesis since mice made deficient of these effectors by thymectomy, irradiation and bone marrow reconstitution fail to develop myocarditis despite high virus titers in the heart [37]. To demonstrate a role for T cells in mediating sex differences in CVB3 pathogenesis, WT male mice were injected on days -3 and +1 relative to infection with 100 μg of monoclonal IgG isotype immunoglobulin or anti-CD3, which depleted > 93% of CD3+ T cells in the animals. T cell deficient male mice developed less myocarditis compared to immunoglobulin treated males (Figure 2; t test *P *< 0.05). T cell depletion had no significant effect on cardiac virus titers confirming earlier studies that CVB3 clearance during primary infection is not dependent on T cell responses [40].

**Figure 3:**
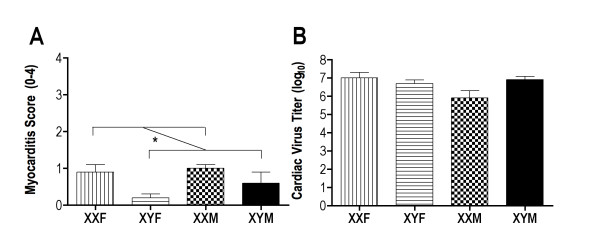
**Myocarditis and cardiac virus titers in four core genotype (FCG) mice infected with coxsackievirus B3 (CVB3)**. FCG mice were gonadectomized (Gdx) and infected with 100 plaque-forming units (PFU) CVB3. Surviving mice were killed 7 days after infection and evaluated for **(a) **myocarditis using a 0-4 histology scale and for **(b) **cardiac virus titer (PFU log10/g tissue). Results represent mean ± SEM of 10-13 mice/group. *Significantly different at *P *< 0.05.

**Figure 4:**
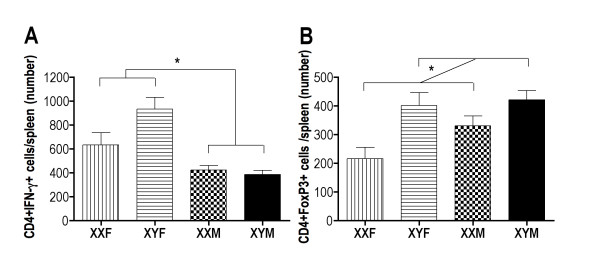
**T helper 1 (Th1) and T regulatory (Treg) cell response in coxsackievirus B3 (CVB3)-infected four core genotype (FCG) mice**. Spleen cells from FCG mice represented in Figure 3 were labeled with antibody to CD4, washed, fixed with 2% paraformaldehyde, permeabilized and labeled intracellularly with antibody to either **(a) **interferon (IFN)γ (Th1 cells) or **(b) **forkhead box P3 (FoxP3) (Treg cells). Results represent the mean ± SEM number of indicated cells/spleen. *Significantly different at *P *< 0.05.

The possibility that sex chromosome complement influences CVB3-induced myocarditis was investigated by infecting Gdx FCG mice and evaluating myocarditis and virus titers 7 days post infection (Figure 3). Myocarditis scores showed a significant effect of sex chromosome complement, in which XX mice developed significantly more myocarditis than XY mice, irrespective of their gonadal sex (two-way ANOVA sex chromosome complement effect *P *< 0.009). There were no differences in cardiac virus among the FCG mice. To evaluate the mechanism by which sex chromosome complement impacts CVB3 myocarditis, spleen cells from FCG mice were isolated and evaluated by flow cytometry for CD4+IFNγ+ (T helper 1 (Th1)), CD4+FoxP3+ (T regulatory) or CD4+IL-4+ (Th2) cell responses. No significant difference in Th2 cell responses was observed among the FCG mice (data not shown). Gonadal females had significantly greater Th1 cell responses than gonadal male mice, regardless of their sex chromosome complement (Figure 4a; two-way ANOVA gonadal sex effect *P *< 0.0006). In contrast, higher T regulatory cell numbers were observed at day 7 post infection in XY than XX mice, irrespective of gonadal sex (Figure 4b; two-way ANOVA sex chromosome complement effect *P *< 0.006). Taken together, these data illustrate that both gonadal hormones and sex chromosomes contribute to CVB3 pathogenicity.

### Sex chromosome complement does not impact susceptibility to IAV infection

**Figure 5:**
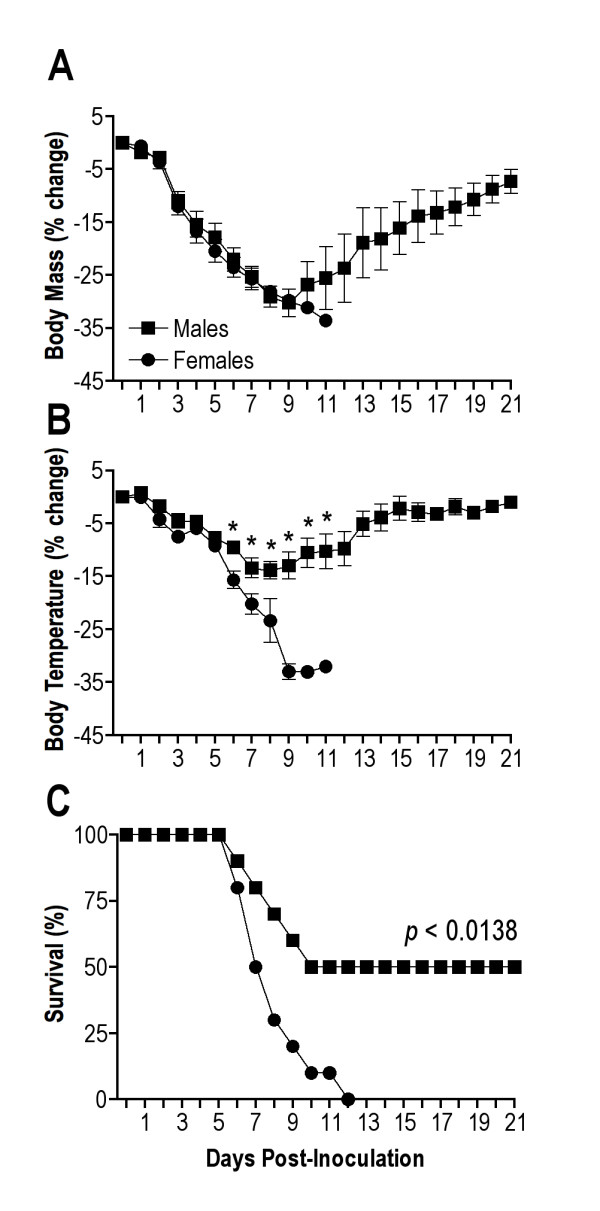
**Morbidity and mortality from influenza A virus (IAV) infection in gonadally-intact male and female C57BL/6 mice**. Average (± SEM) change in body mass **(a) **and rectal temperature **(b) **from baseline (that is, day 0) following inoculation with 10^2 ^50% tissue culture infective dose (TCID_50_) of mouse-adapted IAV, A/Puerto Rico/8/1934 (PR8). The proportion of wild-type (WT) male and female mice surviving PR8 infection during the 21 days post inoculation is shown in **(c)**. Each group consisted of ten mice run in a series of two replications. *Significantly different at *P *< 0.05.

Sex biases also exist in response to IAV infection [31,32,41]. To begin our analyses, we inoculated male and female C57BL/6 mice with IAV and morbidity and mortality were evaluated over a 21-day period in gonadally intact WT mice that were older than in previous studies [32] (that is, animals were 18-22 weeks of age to be age matched to FCG mice). Although males and females lost a similar percentage of body mass, females experienced a greater reduction in rectal temperature than males during infection (Figure 5a, b; MANOVA sex × day interaction *P *< 0.0001). Survival was also significantly reduced among females, in which fewer females survived infection and females died sooner than their male counterparts (Figure 5c; log rank *P *< 0.05). These data illustrate that sex differences in response to IAV infection are observed among older adult mice, corresponding to the sex differences previously shown in younger animals (that is, animals that were 9-11 weeks of age) [32].

**Figure 6:**
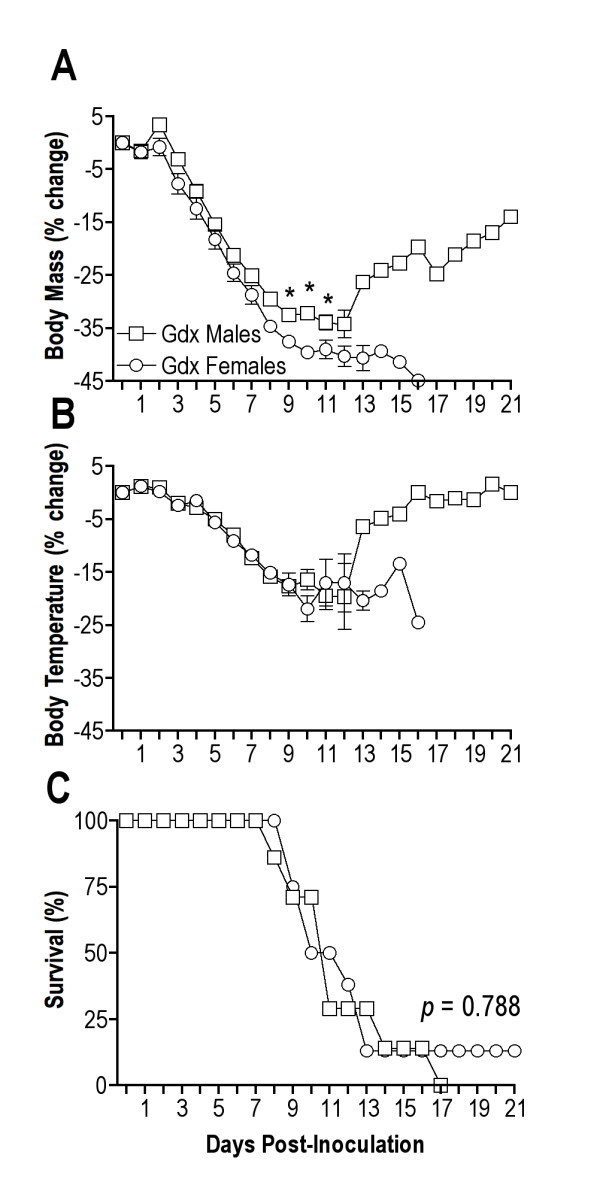
**Morbidity and mortality from influenza A virus (IAV) infection in gonadectomized (Gdx) male and female C57BL/6 mice**. Average (± SEM) change in body mass **(a) **and rectal temperature **(b) **from baseline (that is, day 0) following inoculation with 10^2 ^50% tissue culture infective dose (TCID_50_) of mouse-adapted IAV, A/Puerto Rico/8/1934 (PR8). The proportion of Gdx male and female mice surviving PR8 infection during the 21 days post inoculation is shown in **(c)**. Each group consisted of 7-8 mice run in a series of 2 replications. *Significantly different at *P *< 0.05.

To determine if deprivation of sex steroid hormones could abolish sex differences in morbidity and mortality from IAV infection, WT adult male and female C57BL/6 mice were gonadectomized and infected with IAV. Gdx females lost more weight than Gdx males (Figure 6a; MANOVA treatment × day interaction *P *< 0.01), but showed a similar reduction in rectal temperature as Gdx males (Figure 6b). The proportion of Gdx females and males that died as well as the rate of death were similar following infection (Figure 6c). These data illustrate that sex steroid deprivation reduces, and in some cases eliminates, the sex difference in IAV pathogenesis.

**Figure 7:**
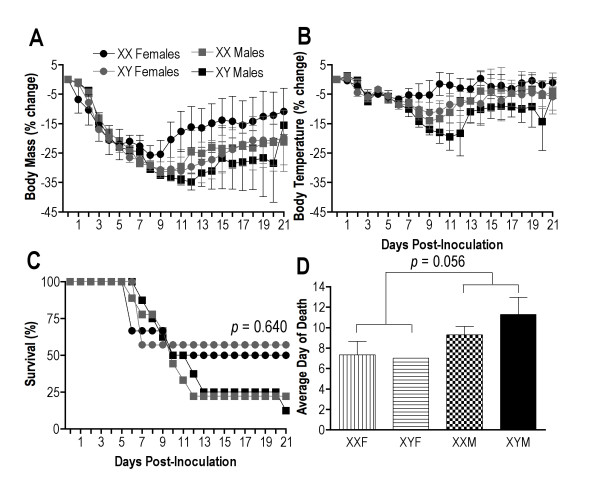
**Morbidity and mortality from influenza A virus (IAV) infection in four core genotype (FCG) mice**. Average (± SEM) change in body mass **(a) **and rectal temperature **(b) **from baseline (that is, day 0) in Gdx XXF, XYF, XXM, and XYM mice following inoculation with 10^2 ^50% tissue culture infective dose (TCID_50_) of mouse-adapted IAV, A/Puerto Rico/8/1934 (PR8). The proportion of mice surviving PR8 infection during the 21 days post inoculation is shown in **(c) **and the average day of death for FCG mice following inoculation with PR8 in **(d)**. Each group consisted of 6-9 mice run in a series of 2 replications.

To examine whether sex chromosome complement affects susceptibility to IAV infection, responses to infection were examined in Gdx FCG mice [33]. All FCG mice lost a similar percentage of body mass and experienced a similar reduction and recovery in rectal temperature during the course of infection (Figure 7a, b). The proportion of mice surviving infection was similar among XXF, XYF, XXM, and XYM mice (Figure 7c). Although not statistically significant, among those animals that died, the average day of death was slightly later for gonadal male (XXM and XYM) than gonadal female (XYF and XXF) mice (Figure 7d; two-way ANOVA gonadal sex effect *p *= 0.056). Taken together, these data suggest that sex chromosome complement does not affect susceptibility to IAV in C57BL/6J mice.

## Discussion

Sex differences in response to viral infections are well documented [1]. In many cases, these differences are modulated by the effects of gonadal hormones, including testosterone and estradiol, on immune responses to infection [1]. This communication confirms an important role for testicular and ovarian secretions in the response to viral infection, and also shows that sex chromosome complement impacts the pathogenicity of CVB3 but not IAV infections in mice.

Host immunity can play a significant role in either recovery or pathogenesis from both picornavirus and influenza virus infections. For picornaviruses, host T cell immunity is particularly detrimental and in CVB3 myocarditis [42], Theiler's virus encephamyelitis [43], and encephalomyocarditis virus-induced diabetes [44], virus infection induces autoimmunity to tissue antigens by antigenic mimicry or epitope spreading. Autoimmune effectors rather than direct virus cytopathic effects are the dominant mechanism of morbidity and mortality in response to these viruses. T regulatory cell activity abrogates autoimmune T cell responses, morbidity and mortality following picornavirus infection [45]. In the present study, activation of T regulatory cells was increased in Gdx XYF and XYM mice compared to Gdx XXM and XXF mice, resulting in preferential protection of these mice from CVB3. Previous studies have linked activation of FoxP3+ T regulatory cells with resistance to CVB3 induced myocarditis, which is consistent with the current finding [39,45]. *Foxp3*, the T regulatory cell transcriptional factor, is encoded on the X chromosome. It is therefore highly probable that the increased T regulatory cell response in Gdx XY mice will explain the increased resistance of these animals to CVB3 induced disease, although this will need to be confirmed by depletion of these cells prior to CVB3 infection. The major question that remains is why the XY sex chromosome complement would give rise to enhanced *Foxp3 *expression when this gene is present on the X chromosome. It is unlikely that the XY sex chromosome complement is directly affecting expression of the *Foxp3 *gene, but may establish conditions that are favorable to T regulatory cell activation.

The dominance of CVB3-induced autoimmune pathogenesis is also shown by the disassociation between myocarditis and cardiac virus titers. T cell depletion reduces cardiac injury but has no effect on virus titers in the heart. If cardiac injury resulted from direct virus infection and replication, then T cell depleted male mice would not be protected when virus load in the myocardium remained high. The inability of T cells to promote CVB3 clearance was initially shown in 1974 using both athymic (nude) and thymectomized, irradiated, bone marrow reconstituted animals [40]. Since then, various studies have demonstrated that T cell independent antibodies are rapidly induced by picornavirus infections, including CVB3 and foot-and-mouth disease virus, which, along with macrophages, are the primary mediators of virus clearance [40,46,47].

Protection against IAV infection represents a delicate balance between immune responses protecting versus causing pathology in the host. Immediately following infection, proinflammatory responses, including production of cytokines (for example, TNFα, IL-6, and IL-1β) and chemokines (for example, chemokine (C-C motif) ligand 2 (CCL2) and CCL3), are initiated by macrophages, dendritic cells, and epithelial cells in the respiratory tract, which activate humoral and cell-mediated immune responses to promote virus clearance and protection of the host from subsequent infection. There is, however, growing evidence that these early proinflammatory events can lead to severe disease and even death through a process termed 'immunopathology' [48]. Immune memory and long-term protective immunity against IAV is mediated by pre-existing antibodies as well as memory B and T cells [49]. Young adult female mice produce significantly higher proinflammatory cytokine and chemokine responses and experience greater morbidity and mortality during IAV infection than males, which appears to involve the effects of infection on circulating levels of estradiol [32]. Influenza A virus infection of female mice disrupts reproductive function resulting in persistently low levels of estradiol and progesterone, heightened proinflammatory responses, and reduced rates of survival [32]. Consequently, the outcome of IAV infection is severe for both gonadally-intact and Gdx female mice and exogenous administration of estradiol to Gdx females significantly reduces the induction of proinflammatory responses and increases rates of survival [32]. Taken together, our previous data combined with data from the current study illustrate that gonadal secretions, but not sex chromosome complement, play a role in modulating responses to IAV infection.

Gonadectomy of female mice substantially increased pathogenicity in females infected with CVB3, but had little effect in IAV infected females. In males, Gdx had the opposite effect, with Gdx protecting CVB3 infected males, but increasing pathogenesis in IAV infected Gdx males. A key difference between IAV and CVB3 infections is in the crucial role of adaptive immunity to clear influenza virus [50,51], whereas T lymphocytes and virus neutralizing antibodies are of limited value for elimination of picornaviruses [40,47]. This difference in the requirement for adaptive immune responses for recovery from IAV and CVB3 likely contributes to why Gdx is protective against CVB3 and promotes pathogenicity of IAV infection.

The response of FCG mice to infection with CVB3 and the development of CVB3-induced myocarditis in XX animals is similar to the responses of FCG mice in experimental models of EAE and lupus, in which the XX sex chromosome complement produces significantly more severe disease than the XY complement [27]. In EAE and lupus models, XX mice had lower levels of Th2 cytokines than XY mice. Consequently, Th2-mediated responses promote resistance against EAE [52], whereas Th1 and Th17 cell activation is crucial to EAE severity [53]. In the current study, XY mice developed less severe CVB3-induced myocarditis and had more T regulatory cells in their hearts than XX mice. T regulatory cells inhibit proinflammatory responses through direct cell-cell contact with effector T cells, interaction with antigen presenting cells resulting increased indoleamine-2,3-dioxygenase (IDO) production, and secretion of soluble factors including IL-10 and transforming growth factor (TGF)β [54-56]. The effect of sex chromosome complement on T regulatory cells was not addressed in previous experiments of EAE and lupus in FCG mice [27]; T regulatory cells, however, are associated with resistance against EAE [57]. An inherent deficiency in T regulatory cell activation in individuals either having two X chromosomes or lacking a Y chromosome is consistent with autoimmune diseases that typically show a strong female bias [23,58].

Three genetic differences between XX and XY mice could contribute to a sex chromosome effect. The Y chromosome may encode genes that normally have an XY-specific effect. Secondly, the double dose of X genes in XX mice, relative to XY mice, could cause constitutively higher expression of some X genes [59]. The process of X inactivation greatly reduces the number of X genes that show such sex differences in expression. There are, however, a small number genes that escape X inactivation resulting in meaningful differences in gene expression [60]. Thirdly, X genes that receive a parental imprint might be expressed higher in one sex than the other, because only females receive a paternal X imprint. Further genetic studies are needed to resolve the chromosome of origin of the sex chromosome effects reported here, and the genes, especially those that code for immune-related proteins, that are responsible.

Sex differences in circulating levels of gonadal hormones contribute significantly to sex differences in response to viral infections [1]; there is, however, little evidence that gonadal secretions have lasting effects that are maintained for several weeks after removal of the gonads. These effects, often called 'organizational' effects of gonadal hormones [61], may account for the differences between Gdx males and females in response to CVB3 and IAV infection. Thus, the immune system, like the brain and genitalia, may reflect lifelong sex differences established by sex differences in patterns of gonadal secretions early during development [62]. Alternatively, the differences in gonadal males and females seen in response to CVB3 may be caused by direct effects of *Sry *on non-gonadal tissues.

## Conclusions

Although sex chromosomes can directly influence CVB3 pathogenesis, the dominant sex effect is mediated by gonadal hormones. Gonadally intact WT males are more susceptible to CVB3 myocarditis than gonadally intact WT females. Although gonadectomy reduces male susceptibility, restoring testosterone to Gdx males results in equivalent myocarditis as in intact animals [30], and treating intact males with estradiol inhibits disease [63]. Similarly, gonadectomy reduces the sex difference in susceptibility to IAV in mice. Taken together, the data presented suggest that any effect of sex chromosome complement on responses to viruses is overpowered by the effects of sex hormones on virus infection and immunity. The significance of direct chromosome effects on clinical CVB3 or other viral infections may be minimal in premenopausal women when estrogen levels are high. Examination of immune responses to viruses in older populations, especially among postmenopausal women, may unmask clinically-relevant effects of sex chromosomal genes on viral disease pathogenesis. Clarification of the relative roles of gonadal hormones and sex chromosome effects will be improved once the X or Y genes responsible for sex chromosome effects are identified.

## Competing interests

The authors declare that they have no competing interests.

## Authors' contributions

DR designed and carried out the IAV studies, analyzed and interpreted IAV data, drafted all figures, and contributed to the writing and editing of the manuscript. SK designed the IAV studies, analyzed IAV data, interpreted IAV data, and contributed to drafting the manuscript. SH designed the CVB3 studies, analyzed and interpreted the CVB3 data, and contributed to the writing and editing of the manuscript. MM and BR carried out the CVB3 studies. CT contributed to the interpretation of the CVB3 data. RW and AA provided FCG mice and contributed to interpretation of data and drafting of the manuscript. All authors approved the final manuscript.
